# Genetic Divergence of *Toxoplasma gondii* Strains Associated with Ocular Toxoplasmosis, Brazil

**DOI:** 10.3201/eid1206.060025

**Published:** 2006-06

**Authors:** Asis Khan, Catherine Jordan, Cristina Muccioli, Adriana L. Vallochi, Luiz V. Rizzo, Rubens Belfort, Ricardo W.A. Vitor, Claudio Silveira, L. David Sibley

**Affiliations:** * Washington University School of Medicine, St. Louis, Missouri, USA;; † Paulista School of Medicine, São Paulo, Brazil;; ‡ University of São Paulo, São Paulo, Brazil;; § Federal University of Minas Gerais, Belo Horizonte, Brazil;; ¶ Clinica Silveira, Erechim, Brazil

**Keywords:** PCR-RFLP, genotyping, genetic diversity, intron sequence, SNP, phylogeny, research

## Abstract

Previous studies have shown a high prevalence of toxoplasmosis and the frequent occurrence of ocular disease in Brazil. To identify the genotypes of parasite strains associated with ocular disease, we compared 25 clinical and animal isolates of *Toxoplasma gondii* from Brazil to previously characterized clonal lineages from North America and Europe. Multilocus nested polymerase chain reaction analysis was combined with direct sequencing of a polymorphic intron to classify strains by phylogenetic methods. The genotypes of *T*. *gondii* strains isolated from Brazil were highly divergent when compared to the previously described clonal lineages. Several new predominant genotypes were identified from different regions of Brazil, including 2 small outbreaks attributable to foodborne or waterborne infection. These findings show that the genetic makeup of *T*. *gondii* is more complex than previously recognized and suggest that unique or divergent genotypes may contribute to different clinical outcomes of toxoplasmosis in different localities.

## Abstract 2

Brazilian strains of *T*. *gondii* differ from lineages in North America and Europe; these differences may underlie severe ocular disease.

*Toxoplasma gondii* is an obligate intracellular parasite that infects a wide range of warm-blooded vertebrates and causes disease in agricultural animals and humans (*1*). *T*. *gondii* has a complex life cycle that includes an asexual cycle and sexual cycle; the asexual cycle occurs in a wide range of intermediate hosts, and the sexual cycle occurs exclusively in feline hosts, which shed infectious oocysts in their feces (*1*). *T*. *gondii* is mainly transmitted by ingesting cysts contained within tissues of a chronically infected host or by ingesting sporulated oocysts from fecally contaminated food or water (*2*). *T. gondii* is an influential foodborne pathogen in the United States (*3*) and a frequent cause of waterborne infection in parts of Brazil (*4**,**5*).

Despite having a sexual phase in its life cycle, the population structure of *T. gondii* is markedly clonal (*6*). Most strains analyzed from North America and Europe belong to 1 of 3 clonal lineages known as types I, II, and III (*7**–**9*). A small number (<5%) of isolates contain different combinations of the same alleles seen in the clonal types, which indicates that recombination occurs infrequently in the wild (*7*). Additionally, strains with more divergent genotypes have been isolated from locations such as French Guiana (*10*).

The serologic prevalence of *T*. *gondii* infection in Brazil includes 50%–80% of the adult population, with the highest values found in northern and southern states (*5*). Waterborne transmission has been implicated in high rates of *T. gondii* seropositivity in northern Rio de Janeiro State (*5*) and in a toxoplasmosis outbreak in Santa Isabel do Ivai in southern Paraná (*4*). High levels of ocular disease are associated with toxoplasmosis in Brazil (*11*). In the southern city of Erechim, Brazil, 184 (17.7%) of 1,042 adults were found to have retinal scars, thought to be caused by toxoplasmosis (*12*). Epidemiologic data indicate that many cases of ocular disease are acquired after birth rather than congenitally (*13**–**15*). Whether the increased prevalence and severity of ocular toxoplasmosis in Brazil are attributable to host or parasite genetic factors or differences in exposure rate is uncertain.

Polymerase chain reaction (PCR)–based typing at the *SAG2* locus has been used previously to suggest that type I strains predominate in Brazilian patients with ocular toxoplasmosis (*16*). While the *SAG2* marker provides accurate genotyping for most strains within the clonal lineages, it cannot detect recombinant strains or those with unusual genotypes (*17*). In fact, the exclusive use of any single locus may misrepresent the genotype of recombinant or unusual genotypes as having a simple genotype. This problem is partially alleviated by multilocus analysis, and random amplified polymorphic DNA (RAPD)–PCR analysis of Brazilian *T. gondii* strains with multiple markers showed that most strains contain both type I and III alleles at different loci (*18*). However, PCR-based markers, such as restriction fragment length polymorphism (RFLP) or RAPD, underestimate the true rate of nucleotide divergence and thus may not accurately classify *T*. *gondii* strains from new regions. For example, a high degree of polymorphism is detected at the *GRA6* locus by sequence analysis (9 allelic sequences among 30 strains), whereas PCR-RFLP analysis differentiates only 3 groups among these same strains (*19*).

We have recently described a sensitive method for multilocus genotyping consisting of nested PCR (nPCR) amplification of 4 different RFLP markers (*SAG2*, *GRA6*, *SAG3*, and *BTUB*). When combined with sequencing of the *UPRT*-1 intron, multilocus nPCR typing provides a robust means to classify strains as having clonal, recombinant, or novel genotypes (*17*). Multilocus nPCR analysis also can detect as few as 5 parasite genomes and thus is applicable to low-volume samples containing few parasites, as is typical of clinical specimens (*17*). In this study, we examined a group of Brazilian *T*. *gondii* strains from animal and human sources, including several outbreaks, to examine the population structure of *T*. *gondii* in Brazil.

## Methods

### Clinical Isolates

Patients were examined at Clinica Silveira, Erechim, Rio Grande do Sul State, by indirect ophthalmoscopy or biomicroscopy by using a slit-lamp microscope. Ocular disease was evaluated on the basis of parameters described previously (*11*). Patient consent was obtained at the time of sample acquisition. Recently acquired toxoplasmosis was confirmed by serologic tests that monitor immunoglobulin G (IgG) and IgM by enzyme-linked immunosorbent assay (Abbott Laboratories, Abbott Park, IL, USA) (*11*). Ocular toxoplasmosis was diagnosed on the basis of recurrent episodes of necrotizing retinochoroiditis. Venous blood was collected before treatment in Vacutainer tubes containing heparin and cells, and serum was separated by centrifugation. The buffy-coat layer was removed, frozen at –20°C, and shipped to Washington University, Saint Louis, for analysis. Blood was also obtained from patients infected during 2 small outbreaks of toxoplasmosis in Santa Vitoria do Palmar, Rio Grande do Sul State, and Agronomica, Santa Catarina State, and processed in a similar manner. Buffy-coat samples were processed by using the DNAeasy tissue extraction kit (Qiagen Inc., Valencia, CA, USA) before PCR analysis.

### Parasite Strains and Tissue Samples

Reference strains consisted of representative members of the 3 clonal lineages originally isolated from human or animal infections in North America and Europe. Reference strains for the clonal types included: 4 type I strains: ENT (ATCC 50850), RH (ATCC 50838), GT1, and VEL (ATCC 50852); 3 type II strains: Me49 (ATCC 50840), DEG (ATCC 50855), and PIH (ATCC 50857); and 3 type III strains: CTG (ATCC 50842), STRL, and VEG (ATCC 50861). In addition, 3 previously reported strains with more divergent genotypes were included: CAST (ATCC 50868), COUG, and MAS (ATCC 50870) (*20*). Parasite strains were grown in human fibroblast cells, harvested after natural egress from host cells and purified as above; cell lysates were prepared for PCR as described previously (*17*). Five Brazilian *T. gondii* strains isolated from Belo Horizonte, Minas Gerais, and one from São Paulo, as described previously (*18*), were also included.

Additional samples of porcine tissue collected from abattoirs in the Erechim region were included (R.N. Belfort, unpub. data). Six samples were analyzed, 2 negative controls and 4 samples that were positive by PCR (data not shown). Tissue samples were extracted with DNAzol, followed by an equal volume of chloroform. Polyacryl carrier (Molecular Research Center, Inc., Cincinnati, OH, USA) was added (5 μL) to the aqueous phase, and DNA was precipitated by adding an equal volume of ethanol and centrifugation at 5,000 × *g* for 10 min.

### Genotyping Isolates by PCR-RFLP

Multilocus nPCR analysis of 4 different loci was based on the markers 5´-*SAG2*, 3´-*SAG2*, *BTUB*, *GRA6*, and *SAG3* (*17*). Amplification was performed as described previously (*20*), and negative controls consisted of sterile, distilled water or proteinase K–treated cell lysate of noninfected host cells. The amplified products were digested with appropriate restriction enzymes for different loci, and the resulting fragments were analyzed by 3% agarose gel electrophoresis, stained with ethidium bromide, and imaged by an Alpha Imager version 5.5 camera (Alpha Innotech Corp., San Leandro, CA, USA).

Restriction fragments were scored visually as present or absent, and a genetic distance matrix was calculated from the proportion of shared restriction sites by using the equation of Nei and Li (*21*). The neighbor-joining method was used to analyze the distance matrix, and dendrograms were generated by using the phylogenetic analysis program PAUP* version 4.0b (*22*). Bootstrap analysis was conducted for 1,000 replicates to obtain confidence estimates for the taxonomic groupings. The conditions were set to distance, neighbor-joining, with mean character differences, and dendrograms were constructed by using the 50% majority rule.

### UPRT-1 Intron Sequences Analysis

Sequence divergence among strains of *T*. *gondii* was determined at the uracil phosphoribosyl transferase (*UPRT*) intron 1 sequence (GenBank accession no. AY143141), as described previously (*17*). Following the previously described nPCR amplification of the *UPRT*-1 intron, a third set of internal primers was used for sequencing: UPRT-1seqF 5´-CTCGTCCTCGTTTTCCTT-3´ and UPRT-1seqR 5´-TGAAAGGAAGCACGTAAAGT-3´. Sequencing was conducted on 3 independent PCR-amplified templates by using BigDye cycle sequencing (Applied Biosystems, Foster City, CA, USA) (conducted by SeqWright DNA Technology Services, Houston, TX, USA). ClustalX/W (*23*) was used to align the sequences for comparison with default settings. After removal of primer sequences, the UPRT-1 intron sequence used for comparison was 467 bp in length. Unrooted phylogenetic comparisons were conducted with distance and parsimony methods by using PAUP*4.0b (*22*). The conditions were set to distance (mean character difference, minimal evolution, negative branches = 0), and 1,000 bootstrap replicates were performed by using the BioNeighbor-Joining algorithm. Alternatively, parsimony analysis was conducted by heuristic stepwise searching, with bootstrapping for >1,000 replicates. Consensus trees were drawn with an arbitrary root according to the bootstrap 50% majority rule.

## Results

**Figure 1:**
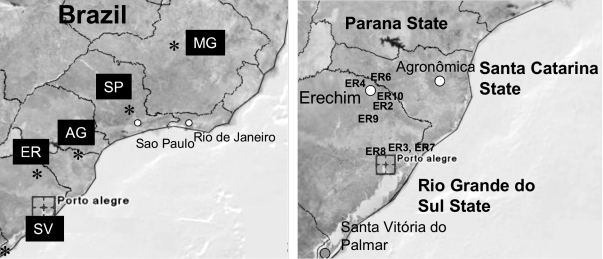
Location of samples obtained from Brazil. A) Samples were collected from Belo Horizonte, Minas Gerais (MG), Erechim City (ER), São Paulo (SP), Agronomica (AG), and Santa Vitttoria do Palmar (SV) (abbreviations as found in the Table). B) Clinical samples were collected from Erechim, the surrounding region (numbered as in the Table), and from 2 outbreaks in AG and SV.

Brazil has a high prevalence of ocular toxoplasmosis, and many of these cases are recurrent and serious in nature (*11**,**12*). This situation prompted us to consider whether sampling patient blood might allow diagnosis of recent (acute) or recurrent infection by direct PCR amplification. Blood was collected from 77 patients seen at the Clinica Silveira, Erechim, Brazil, from 2003 to 2005, and the buffy-coat that contained leukocytes was separated by centrifugation and used for analysis. Nested PCR analysis of these samples by using the *SAG3* gene showed that 11 of 77 were positive, including 6 patients with acute disease and 5 patients with recurrent disease (Table) (locations of the patients with positive samples are shown in Figure 1). We also analyzed several sets of samples from 2 small outbreaks of acute toxoplasmosis. The first in Santa Vitoria do Palmar consisted of 10 persons from a single family that shared a meal of home-cured sausage that contained pork. Symptoms in infected persons included lymphadenitis, myalgia, fever, headache, and sweating. One of these patients, a 53-year-old woman, had severe retinochoroiditis. Only a single sample from these 10 persons was positive by *SAG3* nPCR analysis of buffy-coat cells. A second outbreak consisted of 8 infected persons from Agronomica, a town of ≈4,000 residents located 200 km from Florianopolis. These 8 persons shared the same source of nontreated water in a common neighborhood, and their illness was likely caused by waterborne infection. Three of these persons had positive results by *SAG3* nPCR.

**Table:** Genotypes of *Toxoplasma gondii* in human ocular toxoplasmosis samples from Brazil*

Strain name	Type of sample	Source	Locus							
			5´-SAG2†	3´-SAG2‡	BTUB§	SAG3	GRA6	Genotype	Reference¶	Location
ENT	C#	Human	1**	1	1,1	1	1	I	ENT	F
RH	C	Human	1	1	1,1	1	1	I	RH	USA-OH
GT1	C	Goat	1	1	1,1	1	1	I	GT1	USA-MD
VEL	C	Human	1	1	1,1	1	1	I	VEL	USA-CA
Me49	C	Sheep	1	2	2,2	2	2	II	Me49	USA-CA
DEG	C	Human	1	2	2,2	2	2	II	DEG	F
PIH	C	Human	1	2	2,2	2	2	II	PIH	USA-CA
CTG	C	Cat	2	1	2,1	3	3	III	CTG	USA-NIH
STRL	C	Human	2	1	2,1	3	3	III	STRL	USA-CA
VEG	C	Human	2	1	2,1	3	3	III	VEG	USA-CA
CAST	C	Human	1	1	1,1	1	1	I	CAST	USA-CA
COUG	C	Cougar	1	2	2,2	3	2	I/II/III	COUG	CAN-BC
MAS	C	Human	1	1	2,1	3	3	I/III	MAS	F
PBR	C	Dog	1	1	1,1	3	3	I/III	MG1	SP
D3	C	Dog	1	1	2,1	3	3	I/III	MG2	MG
CH1	C	Chicken	2	1	2,1	3	3	III	MG3	MG
CH2	C	Chicken	2	1	2,1	3	3	III	MG4	MG
EFP	C	Human	1	1	2,1	3	3	I/III	MG5	MG
SAF	C	Human	1	1	2,1	3	3	I/III	MG6	MG
6T	A††	Porcine	1	1	2,1	3	3	I/III	P1	EC
7T	A	Porcine	1	1	2,1	3	3	I/III	P2	EC
8T	A	Porcine	1	1	2,1	3	2	I/II/III	P3	EC
9T	A	Porcine	1	1	2,1	3	3	I/III	P4	EC
2147	Cl‡‡	Recurrent ocular	1	1	1,1	2	1	I/II	ER1	ER
2324	Cl	Acute ocular	1	1	1,1	2	1	I/II	ER2	ER
2296	Cl	Acute ocular	1	1	1,1	2	1	I/II	ER3	ER
2323	Cl	Acute ocular	1	1	1,1	2	1	I/II	ER4	EC
2434	Cl	Recurrent ocular	1	1	1,1	2	1	I/II	ER5	ER
2325	Cl	Acute ocular	1	1	1,1	3	1	I/III	ER6	ER
2566	Cl	Recurrent ocular	1	2	1,1	3	–§§	I/II/III	ER7	ER
2583	Cl	Acute ocular	1	1	1,1	1	–	I	ER8	ER
2612	Cl	Recurrent ocular	1	–	1,1	2	–	I/II	ER9	ER
2670	Cl	Recurrent ocular	1	–	1,1	2	–	I/II	ER10	EC
2728	Cl	Acute ocular	1	1	1,1	1	–	I	ER11	EC
2694	Cl	Outbreak	1	2	1,1	3	–	I/II/III	SV	SV
2712	Cl	Outbreak	1	–	1,1	2	–	I/II	AG1	AG
2717	Cl	Outbreak	1	–	1,1	1	–	I	AG2	AG
2719	Cl	Outbreak	1	1	1,1	2	1	I/II	AG3	AG

*C, culture; A, animal; Cl, clinical; F, France; CA, California; NIH, National Institutes of Health; CAN-BC, British Columbia, Canada; SP, São Paulo; MG, Belo Horizonte, Minas Gerais; EC, Erechim City; ER, Erechim region; SV, Santa Vitttoria do Palmar; AG, Agronomica. †Genotypes I and II are the same. ‡Genotypes I and III are the same. §Alleles represent *Bsi*EI and *Taq*I, respectively. ¶As referred in figures. #Culture strains used as reference. **Alleles defined by pattern in type I strain = 1, second allele = 2; allele 3 is defined by the presence of a second biallelic polymorphism. ††Meat tissue samples, primary source. ‡‡Human ocular toxoplasmosis. §§Refers to negative amplification product.

**Figure 2:**
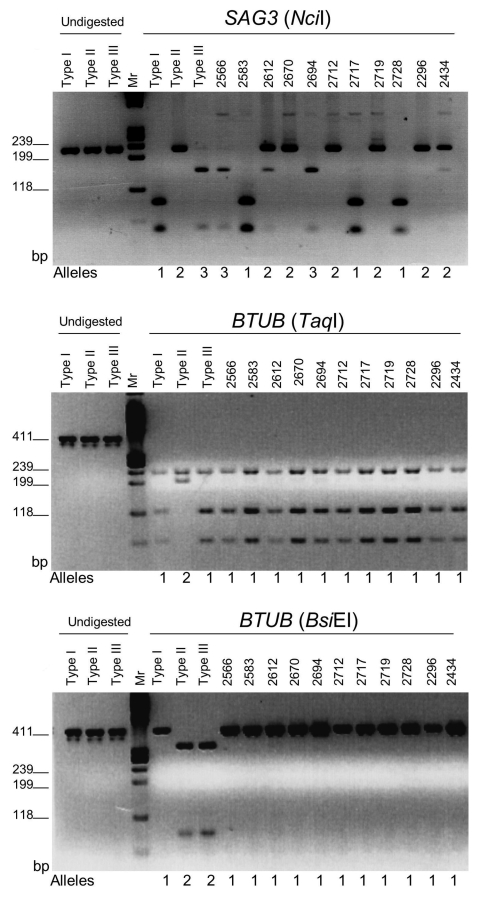
Polymerase chain reaction–restriction fragment length polymorphism (PCR-RFLP) analyses of clinical isolates from Brazil compared to analyses of clonal strains. Shown are the PCR markers *SAG3* and *BTUB*, with their respective restriction digests. Alleles are designated below each figure panel and match those given in the Table. Agarose gel electrophoresis of undigested and restriction digested products for type stained (type I RH, type II Me49, type III CTG). Products were resolved on 3% agarose gels strains with ethidium bromide. Mr refers to size markers from φX174 digested with *Hae*III.

To determine the genotype of *T*. *gondii* strains present in clinical samples from Brazil, multilocus nPCR was conducted by using 4 independent markers, *SAG2*, *BTUB*, *GRA6*, and *SAG3*, as described previously (*17*). The 15 clinical samples found to be positive for *SAG3* were genotyped for most of these markers, although in some cases, insufficient material was available to type all markers (Table). We compared these isolates to strains previously characterized from Brazil and to the clonal lineages common in North America and Europe. In total, 38 strains were subjected to multilocus nPCR analysis, and after restriction digestion and gel electrophoresis of the products, the strains were classified on the basis of the alleles present relative to the reference strains (Table, Figure 2) (*17*). Three of the ocular toxoplasmosis samples carried alleles characteristic of type I strains at 3 or more independent markers, and 2 Brazilian chicken strains possessed alleles typical of type III strains at all loci. All of the remaining Brazilian samples had genotypes consisting of different combinations of alleles seen in the clonal types. Nine clinical samples, including samples from Agronomica from 2 outbreaks, possessed the same profile that consisted of alleles typical of type I and type II lineages. The nPCR assay used here can detect both alleles equally well for all the makers studied, yet in no case were 2 alleles detected at a single locus within a single strain (data not shown). Consequently, the genotypes observed in Brazilian isolates cannot be explained by "mixtures" of >1 strain in a given patient or sample.

**Figure 3:**
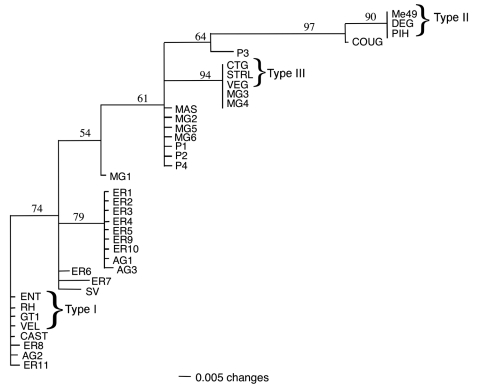
Neighbor-joining phylogram of 38 Toxoplasma gondii strains derived from polymerase chain reaction–restriction fragment length polymorphism typing at loci (SAG2, SAG3, GRA6, and BTUB). Distances were calculated according to Nei and Li (*21*) and the distance matrix analyzed using the phylogenetic analysis program PAUP*4.0b to generate an unrooted phylogram (*22*). The numbers on the branches indicate the bootstrap values (1,000 replicates). Strain designations are shown in the Table.

The percentage of nucleotide divergence between strains was estimated from the proportion of shared restriction sites at each locus, and a distance matrix was used to construct a dendrogram by using neighbor-joining analysis (Figure 3). All the strains belonging to type II were clustered together with a high degree of confidence. All type III strains and 2 chicken strains from Brazil were grouped together with a similarly high confidence. The 4 type I reference strains and 3 human Brazilian clinical isolates (ER8, ER11, and AG2) were clustered together. However, most Brazilian *T*. *gondii* strains were clustered into 2 new groups that were intermediate between types I and III. These results suggest the presence of at least 2 additional haplotypes that are prevalent in Brazil and which differ from North America and European lineages. MAS, which was isolated from a patient with a congenital case of toxoplasmosis in France, clustered with 1 of these Brazilian haplotypes.

**Figure 4:**
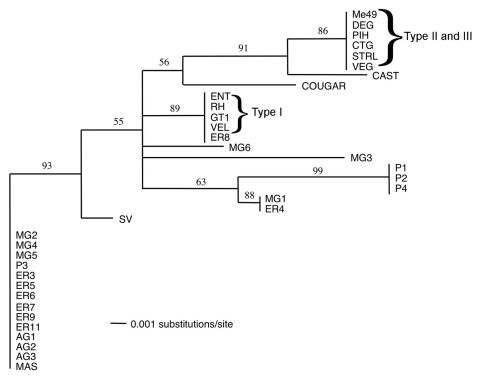
A phylogram of 35 Toxoplasma gondii strains was constructed from a Clustal alignment of UPRT-1 intron sequences using the phylogenetic analysis program PAUP*4.0b (*22*). The BioNeighbor-Joining algorithm was used to determine the divergence distance among different strains and generate an unrooted phylogram. Consensus trees were bootstrapped for 1,000 replicates and drawn with an arbitrary root according to the 50% majority rule. Strain designations are shown in the Table. A complete listing of intron sequences is found in Figure A1.

**Figure A1:**
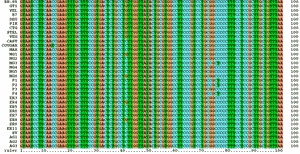
Complete listing of UPRT-1 intron sequences from 35 Toxoplasma gondii strains aligned by using ClustalX. Download PDF

To more accurately assess genetic divergence, we characterized the strains by *UPRT*-1 intron sequencing, a method that is highly sensitive for detecting divergent strains (*17*). *UPRT*-1 intron sequences from 35 strains (1 clinical sample was not available in sufficient quantity for analysis, and 2 samples gave unsatisfactory sequence quality) were aligned by using Clustal X (Figure A1), and the relative divergence of different Brazilian strains was determined by phylogenetic comparison. The results of parsimony and distance analysis were similar and the neighbor-joining distance analysis is shown in Figure 4. Because of the strongly biallelic pattern of *T*. *gondii*, types II and III are identical at the *UPRT* locus, while type I possess a unique haplotype distinguished by 6 single nucleotide polymorphisms (*17**,**20*). Most Brazilian *T*. *gondii* strains (13 of 22) shared a new allele that was distinguished by 6 additional polymorphisms not seen in the clonal lineages (Figure A1). This new Brazilian allele was also shared by the previously characterized divergent strain MAS (Figure 4). Additionally, 3 outbreak strains (AG1, AG2, AG3) from Agronomica and 1 strain each from chickens (MG4) and pigs (P3) were found in this group that otherwise contained a majority of ocular toxoplasmosis isolates from the Erechim region. Other strains from Brazil contained equally divergent but unique alleles that in some cases formed smaller groups (i.e., P1, P2, P4 and MG1, ER4) (Figure 4). Only a single Brazilian strain (ER8) contained a haplotype characteristic of 1 of the clonal lineages, and this strain was identical to the type I lineage in both the PCR-RFLP and *UPRT*-1 intron trees.

## Discussion

*T*. *gondii* is highly prevalent in Brazil, where human infection is associated with an unusually high occurrence of ocular disease in some locations. We examined the genotype of *T*. *gondii* strains collected from a variety of sources in southern Brazil. Included in this study were a group of patients seen at an eye clinic in Erechim, a region known for high levels of ocular toxoplasmosis (*11*). We also examined strains from several small outbreaks from nearby regions and compared these strains to animal isolates from Erechim and the more central region of Minas Gerais. Multilocus PCR-RFLP and sequenced-based analysis showed that they differ substantially from the previously described clonal lineages and instead define several new haplotypes that appear to be predominant in Brazil. The abundance of genotypes that do not fit the conventional classification shows the global pattern of *T*. *gondii* population structure to be more complex than previously thought. These findings have implications for the transmission of *T*. *gondii* as a waterborne and foodborne human pathogen and for studies on the role of genetic composition in virulence, pathogenesis, and life cycle dynamics.

Most human infections of *T*. *gondii* are not clinically severe and progress rapidly to a chronic state that is characterized by semidormant tissue cysts (*2**,**24*). During the chronic infection, parasites are generally not found in circulation, and obtaining parasites without performing invasive procedures such as tissue biopsy is relatively difficult. Previous reports have suggested that parasites may be found circulating in blood during reactivation of toxoplasmosis in AIDS patients (*25**,**26*). Our studies show that by using highly sensitive and specific nPCR, small numbers of parasites may be detected in circulating blood from some patients with either acute-onset or recurrent ocular toxoplasmosis. False-positive PCR amplification did not appear to be a substantial problem, as shown by consistently negative results for water and host-cell-only samples and the fact that the genotypes of clinical strains did not resemble common laboratory strains that would be the likely source of any contamination.

Genotyping *T*. *gondii* strains found in clinical and animal samples from Brazil showed that all strains except 1 (ER8) had different genotypes from clonal lineages that predominate in North America and Europe. When analyzed by multilocus PCR-RFLP, these new South American genotypes initially appeared to be composed of different combinations of alleles seen in the clonal types, similar to findings of a previous report from Brazil (*18*). This pattern could indicate that Brazilian strains of *T*. *gondii* undergo more frequent sexual recombination, resulting in mixed genotypes. However, the true extent of sequence divergence is not captured by multilocus RFLP analysis. We have previously shown that direct sequencing of introns from housekeeping genes provides a more accurate picture of sequence divergence (*17**,**20*). Introns are also likely to be selectively neutral and therefore well suited for phylogenetic comparisons (*27*). In the present study, when the *UPRT*-1 intron sequence was compared, all strains from Brazil except 1 (ER8) had multiple additional polymorphisms not seen in the clonal lineages. This locus indicates a low genetic diversity in *T*. *gondii* strains in Brazil, although they include genotype(s) uncommon in North America and Europe. Both the RFLP and intron analysis indicate several predominant haplotypes in Brazil, along with less common unique genotypes. Further studies will be necessary to define the population structure of *T*. *gondii* in Brazil and other South American locations.

The high seropositivity to *T*. *gondii* (*11**,**28**,**29*), combined with unusually high levels of ocular disease in some regions, shows that toxoplasmosis is a notable health problem in Brazil. Previous studies have shown a high prevalence of *T*. *gondii* in food animals such as pigs (*30*) and chickens (*31*) and in companion animals such as dogs (*32*) and cats (*33*). Although companion animals are not typically a source of human infection, the high prevalence in these species indicates a high level of transmission in Brazil. A recent survey of pig samples obtained from abattoirs in the Erechim region indicated a high prevalence of *T*. *gondii* (35%–66% positive by PCR) (R.N. Belfort, unpub. data). Previous studies have shown a high level of recurrent ocular disease from this region, where 17.7% adults were found to have retinal scars, likely due to toxoplasmosis (*11**,**12*). In addition, drinking unfiltered water has been associated with an increased risk of *T*. *gondii* seropositivity in north Rio de Janeiro State, Brazil (*5*).

Collectively, these epidemiologic features suggest that infection with *T*. *gondii* in Brazil is more likely to lead to serious ocular disease, even in otherwise healthy persons. The extent to which host genetics, immune status, and exposure rate contribute to this pattern is unknown. However, an obvious difference is the markedly different genetic makeup of Brazilian strains of *T*. *gondii*. Previous studies of recurrent ocular toxoplasmosis in patients in the United States have also shown an elevated frequency of unusual genotypes (*34*). Although small animal models have been used for evaluating virulence traits of *T*. *gondii* strains (*6*), comparisons have not yet been made between North American and South American strains in terms of their potential to cause ocular disease.

We have previously advocated using SAG2 for genotyping *T*. *gondii* strains, since it is capable of distinguishing all 3 clonal genotypes at a single locus (*35*). This approach works well in North America and Europe, where the 3 major lineages predominate because of extreme linkage disequilibrium (*7*). Our current findings indicate that most strains from Brazil do not fit the clonal pattern seen in North America. Additionally, *T*. *gondii* strains isolated from French Guiana are also genetically distinct from the clonal lineages seen in North America (*10*). Consequently, studies that rely solely on *SAG2* typing will necessarily underrepresent the true genetic divergence in many regions. *SAG2* typing has been used for genotyping *T*. *gondii* isolates from various animals in Brazil (*30**–**33**,**36*), other parts of South America (*37**,**38*), and Africa (*39**,**40*). Researchers also recently suggested that strains associated with an outbreak of waterborne toxoplasmosis in Paraná, Brazil, were type I strains, based solely on genotyping with the SAG2 marker (*4*). However, analyses based solely on SAG2 almost certainly underestimate the genetic diversity of *T*. *gondii* in these regions. Further strain comparisons based on a wider set of sequence-based markers will be necessary to define the global population structure of *T*. *gondii* and to resolve the relationships between major strain types seen in different regions. Establishing the population structure of *T*. *gondii* is highly relevant to transmission dynamics because the suggestion has been made that recently derived clonal lineages arose through a process of recombination that led to enhanced asexual oral transmission (*20*). Whether other, more divergent strains also express this trait and to what extent their genetic makeup contributes to transmission are highly relevant to understanding the pathogenesis of toxoplasmosis.
